# An Introduction to the Special Issue “Protein Glycation in Food, Nutrition, Health and Disease”

**DOI:** 10.3390/ijms232113053

**Published:** 2022-10-27

**Authors:** Naila Rabbani, Paul J. Thornalley

**Affiliations:** 1 Department of Basic Medical Science, College of Medicine, Qatar University Health, Qatar University, Doha P.O. Box 2713, Qatar; 2 Diabetes Research Center, Qatar Biomedical Research Institute, Hamad Bin Khalifa University, Qatar Foundation, Doha P.O. Box 34110, Qatar

On 20–24 September 2021, leading researchers in the field of glycation met online at the 14th International Symposium on the Maillard Reaction (IMARS-14), hosted by the authors of this introductory editorial, who are from Doha, Qatar. The conference was well-attended, with presentations from 22 countries in the Middle East, Asia, Europe, and North, Central, and South America. The keynote speaker was Lasker Laureate Professor Kazutoshi Mori, speaking on the unfolded protein response, and there were sessions on: glycation in obesity, diabetes, and diabetic complications; glycation in food; glycation through the life course—from maternal bonding to aging; glycation in plants—physiology, function, and food security; glycation in the COVID-19 response; glycation analytics and chemistry; glycation in kidney disease, cancer, and mental health; glycation-related imaging, diagnostic algorithms, and therapeutics; and methods and models in glycation research. The *IJMS* Special Issue on “Protein Glycation in Food, Nutrition, Health and Disease” brings together papers on some of the key issues covered in the virtual conference. We also introduced to the conference the “Doha Glycation declaration”—an outline of areas of glycation research which will likely be fruitful to pursue to future studies, which gained widespread support. In this editorial, we give a brief introduction to this Special Issue, the development of glycation research, the papers presented, and the declaration. We hope that you enjoy this Special Issue on glycation research.

Established in 2005, the International Maillard Reaction Society (IMARS) brings together scientists, clinicians, and technologists involved in the field of research on glycation reactions in foods, biology, and medicine. It promotes research on glycation, the Maillard reaction, and its many and diverse applications. It has organized regular international conferences on this theme since 1979. IMARS is the professional society for the glycation research community, the “home of glycation research” [1].

Glycation is an omnipresent non-enzymatic modification of proteins, DNA, and basic phospholipids [2]. It is a complex process of multiple sequential and parallel reactions called the Maillard reaction. Many advances have been made in glycation research, and some key contributions, as highlighted in the IMARS Presidents’ opening address at IMARS-14, are given in Table 1. The most widely studied glycating agent is glucose, with investigations of the reactive dicarbonyl metabolite methylglyoxal (MG) becoming of increasing interest [3]. Widely studied glycation adducts include fructosamine derivatives—early stage glycation adducts derived from glucose (particularly fructosamine derivatives of hemoglobin in glycated hemoglobin HbA_1c_ or A1C)—which have been recently reviewed [4], and advanced glycation end products (AGEs)—particularly MG-derived AGE, hydroimidazolone MG-H1 [5], fructosamine degradation product, N_ε_-carboxymethyl-lysine (CML) [6], and the trace-level pentose-derived crosslink and intense fluorophore pentosidine [7]. Our own research, spanning over 40 years, has focused mainly on MG—its formation, metabolism by glyoxalase 1 (Glo1) of the glyoxalase system, and as a precursor of AGEs. MG is a precursor of the quantitatively major protein and nucleotide AGEs, arginine-derived MG-H1 and deoxyguanosine-derived imidazopurinone MGdG, respectively [8,9]. Increased MG is a contributing factor to aging and pathological mechanisms of disease, and may be pharmacologically modified for therapeutic outcomes [10]. In this Special Issue, Nagai and coworkers found that MG-H1 was also formed in albumin glycated by ribose in processes inhibited by the chelation of redox active metal ions [11]. This may be mediated by Namiki pathway fragmentations of the ribose moiety, similar to those found in the formation of MG-H1 in albumin glycated by glucose [12,13].

In biomedical applications, it is well-established that protein glycation impacts physiological function in health, aging, and disease [4,10,14,15]. In food science and nutrition, it is also recognized that protein glycation impacts food, influencing taste, texture, processing, stability, digestibility, nutritional value, and the intestinal microbiome [16,17]. Glycation can be compared with spontaneous oxidative modifications increased in oxidative stress [18]. It is also linked to oxidative stress since some glycation processes are oxidative and some glycating agents, such as MG, are metabolized by the antioxidant glutathione (GSH). Oxidative stress and glycation are thereby linked inextricably [10]. Glycation research now has its own stress nomenclature: “dicarbonyl stress”, defined as the abnormal accumulation of dicarbonyl metabolites, leading to increased protein and DNA modifications, contributing to cell and tissue dysfunction in ageing and disease—in which the accumulation of MG often has an important role [19].

In proteins, glycation occurs on amino acid residues with the highest predicted probabilities of being located in functional domains: arginine and lysine [20]. Therefore, glycation has great potential for functional impairment. Indeed, in the proteome of human subjects, *homo sapiens*, and the higher plant, *Arabidopsis thaliana*, amino acids susceptible to oxidation—cys, met, tyr, and trp—are sparse or negatively enriched in function domains of proteins [21,22], which, if generally applicable, suggests that proteomes tend to be resistant to functional impairment by oxidative damage in oxidative stress but are susceptible to functional impairment by glycation—particularly arginine-directed glycation in dicarbonyl stress. In the formation of hydroimidazolone MG-H1 from an arginine residue, there is a loss of positive charge and an increase in hydrophobicity [23]. This produces protein misfolding and the activation of the unfolded protein response (UPR) [24]. Indeed, there is now increasing evidence that MG-modified proteins may be key physiological substrates of the UPR and exert inflammatory effects through the inositol-requiring enzyme-1α of the UPR [25]. In model dicarbonyl stress we found an increase in ubiquitin ligases that likely catalyze the ubiquitination and degradation of MG-modified proteins; and chaperones themselves are susceptible to MG modification [24,25]. Therefore, dicarbonyl stress likely has a key role in stimulating the UPR and related increased proteolysis as well as low-grade inflammation in both mammals and higher plants.

The glycation of DNA in vivo provides the highest level of damaging adducts formed spontaneously in the genome, and is linked to mutagenesis and cancer. Glo1, which suppresses levels of MG and MGdG, is also a tumor suppressor protein [9,26]. In this Special Issue, Donnellan et al. [27] describe how high levels of exogenous MG impair sister chromatid separation in lymphocytes, which may be related to the tumorigenesis activity of MG and is consistent with the tumor suppressor activity of Glo1 [26]. Glo1 inhibitors have been developed for cancer chemotherapy to induce an increase in cellular MG for cytotoxic effects. The lead compound is a cell-permeable prodrug diester of the Glo1 inhibitor, S-p-bromobenzylglutathione cyclopentyl diester (BBGD) [28]. BBGD antitumor activity was enhanced 60-fold in hypoxia, where increased anerobic glycolysis is associated with the increased formation of MG [29,30]. An increase in cellular MG to cytotoxic levels was also involved in the mechanism of action of multiple classes of clinical antitumor drugs: DNA alkylators—mechlorethamine and mitomycin C; topoisomerase inhibitors—camptothecin, doxorubicin, and etoposide; antitubulins—paclitaxel and vincristine; and an antimetabolite, methotrexate. The overexpression of Glo1 contributed to resistance to the antiproliferative cytotoxic activity of these drugs in vitro, and high Glo1 expression is associated with multidrug resistance in clinical cancer chemotherapy [29,31]. The Glo1 inhibitor was active against multiple types of tumor cell lines—leukemia, non-small-cell lung cancer, colon, central nervous system, melanoma, ovarian, renal, prostate, and breast cancer cell lines—and was most potent against the glioblastoma SNB-19 cell line [32,33]. Recently, BBGD was found to have potent antitumor activity in glioblastoma multiforme tumor-bearing mice [34]. Tumors of this type, with a high expression of Glo1, are likely the most suitable ones for clinical evaluation of BBGD [28,29]

In studies on the mechanism of action of the cytotoxicity induced by MG, a decrease in spliceosomal and ribosomal proteins in addition to increased MG modification of the spliceosomal proteins were involved in the commitment to apoptosis after 6 h of treatment [29]. Independent proteomics studies on the WIL2-NS lymphoblastoid cell line, treated for 24 h with MG, reported herein by Donnellan et al. [35] found evidence of MG modification of glycolytic enzymes: glucose-6-phosphate isomerase, aldolase, triosephosphate isomerase, glyceraldehyde 3-phosphate dehydrogenase, phosphoglycerate kinase, enolase, and pyruvate kinase. Marked advances have been made recently in the proteomics of dicarbonyl stress. We now have proteomic signatures indicative of the MG-induced activation of the UPR [24], activation of ubiquitin ligases [25], and commitment to apoptosis [29] as well as histone proteins [36]; a mass increment on arginine residues of +54 is now included in the PRIDE proteomics identification database [37], reflecting the detection of MG-H1 [5,38,39]. The new finding of the susceptibility of the spliceosome to MG modification [29], corroborated independently by Donnellan et al. [28], identifies the spliceosome as a target of glycation in cytotoxicity in cancer chemotherapy. Interestingly, dicarbonyl stress may also contribute to the dysfunction of the spliceosome in diabetes [40,41], chronic kidney disease [42], and Alzheimer’s disease [43].

Protein glycation has long been proposed to have a role in the development of vascular complications in diabetes. In the Special Issue, there are contributions on the hexokinase-2-linked glycolytic overload initiation of processes leading to dicarbonyl stress in vascular cells in hyperglycemia [44], the physiological consequences of dicarbonyl stress [45], the involvement of the receptor for advanced glycation adducts (RAGE) [46], including the role of AGE-RAGE signalling as a modulator of gut permeability in diabetes [47], and the emergence of Glo1 inducer therapeutics for the prevention of type 2 diabetes and treatment of vascular complications of diabetes [28]. The remarkable emerging role of RAGE in early life nurturing as well as the mother–infant bond and the role of glycation in organelle stress as well as metabolic derangement in kidney disease are also described [48,49]. The clinical diagnostics of glycation is now advancing from the role of A1C and glycated albumin in the assessment of glycemic control in diabetes [4] to the application of machine learning for the development of diagnostic algorithms with combinations of plasma and urinary AGEs as features for the risk prediction of diabetic kidney disease and other clinical conditions [50]. The health benefits of decreasing the clinical exposure of dietary AGEs to the microbiome were tested robustly in the “deAGEing Trial”—a 4-week diet low or high in AGEs in obese subjects. A limited impact on the gut microbial composition was found [51].

**Table 1:** Key events in the history of glycation research—a personal view.

Year	Events in the History of Glycation Research (Investigators and Events)	Ref.
1912	Maillard. Studied the reaction between glucose and glycine on heating—“Maillard reaction”.	[52]
1913	Neuberg, Dakin, and Dudley. Discovery of the glyoxalase system.	[53,54]
1937	Kuhn and Weygand. Formation of N-substituted 1-amino-1-deoxy-2-ketose—“Amadori rearrangement”.	[55]
1953	Hodge. Dehydrated foods: chemistry of browning reactions.	[56]
1960	Kato. 3-Deoxyglucosone and 3-deoxypentosone from the browning reactions of glucose and ribose.	[57]
1969	Rahbar et al. Discovery of glycated hemoglobin.	[58]
1980	Hayashi and Namiki. Fragmentation of the saccharide moiety early in the Maillard reaction.	[59]
1984	Cerami et al. First structure proposed for an advanced glycation end product—AGE.	[60]
1986	Baynes et al. N_ε_-carboxymethyllysine (CML) as a degradation product of fructoselysine.	[6]
1989	Sell and Monnier. Structure elucidation of a senescence crosslink—“Pentosidine”.	[7]
1992	Schmidt et al. Isolation and characterization of receptor of advanced glycation end products—RAGE.	[61]
1992	Lo and Thornalley. Cell-permeable glyoxalase 1 inhibitor with anticancer activity—BBGD.	[32]
1994	Bolton et al. and Freedman et al. Clinical trials of aminoguanidine in diabetic nephropathy—ACTION I and II trials.	[62,63]
1994	Henle et al. Detection and identification of a protein-bound hydroimidazolone—MG-H1.	[64]
2000	Delpierre et al. Identification of a mammalian fructosamine-3-kinase.	[65]
2003	Thornalley et al. Application of LC-MS/MS for the robust quantitative assessment of glycation adducts.	[5]
2008	Morcos et al. Extension of lifespan in nematode Caenorhabditis elegans via the overexpression of glyoxalase 1.	[66]
2016	Thornalley et al. Clinical trial of an optimized glyoxalase 1 inducer.	[67]
2016	Rabbani et al. Application of artificial intelligence machine learning for the development of clinical diagnostic algorithms with features including FL and AGEs (diagnosis and classification of early stage arthritis).	[68]

In focusing on glycation in food, the wealth of glycation adducts present in food are still to be fully characterized, with the benefits and adverse effects of glycation on food remaining continuing interests. Reported herein, Xing and Yaylayan investigated the isomeric diversity of glycated amino acids in the Maillard reaction and found that, in a glucose and glycine model system, monoglycated glycine had equal proportions of Amadori and Schiff’s base forms of early glycation adducts, whereas diglycated glycine was a mixture of the three isomers containing two Schiff’s base adducts, two Amadori adducts, and a Schiff’s base–Amadori adduct combination. Glycation processes were stimulated by a mechanochemical reaction, where samples were prepared through ball milling at an ambient temperature and by hydrothermal reactions by heating the mixture in water [69]. It remains uncertain as to whether dietary dicarbonyl compounds may influence the microbiome, have significant bioavailability, and modify endogenous proteins. Furthermore, 3,4-dideoxyglucosone-3-ene (3,4-DGE) is a reactive dicarbonyl compound and glucose degradation product found in processed foods and medicinal products, such as heat-sterilized dialysis fluids containing glucose as osmolyte [70], and may also be formed endogenously. Audiore et al. [71] describe the reaction of 3,4-DGE with human serum albumin, GSH, and immunoglobulin G under physiological conditions.

There were many other leading contributions to glycation research presented at the IMARS-14 conference, and we thank the presenters for their efforts, insights, and ingenuity.

In looking forward to areas of ongoing and future glycation research likely to produce key advances, we signed and invited conference delegates to sign a declaration, the 2021 Doha Glycation declaration. This stated:

“*We, the undersigned—glycation researchers at IMARS-14, identify the following areas for priority advance in glycation research:*

1. *Research in glycation-related analytical techniques and chemistry*
2. *Research in food processing for safe and nutritious food*
3. *Research in glycation-resistant crops for improved food security in climate change*
4. *Research on clinical diagnostics for improved diagnosis, risk prediction and therapeutic monitoring of health conditions and disease*
5. *Research on therapeutics for improved treatment of disease*—*including COVID-19*”.

The declaration was signed and supported by the conference delegates, and we look forward to key advances in glycation research in this and other areas in the years to come.

## Data Availability

Data sharing is not applicable to this article.

## Abbreviations

AGE, advanced glycation end product; A1C, glycated hemoglobin HbA_1c_; BBGD, S-p-bromobenzylglutathione cyclopentyl diester; CML, N_ε_-carboxymethyl-lysine; 3,4-DGE, 3,4-Dideoxyglucosone-3-ene; FL, N_ε_-fructosyl-lysine; Glo1, glyoxalase 1; GSH, reduced glutathione; IMARS, International Maillard Reaction Society; LC-MS/MS, liquid chromatography–tandem mass spectrometry; MG, methylglyoxal; MGdG, methylglyoxal-derived imidazopurinone, 3-(2’-deoxyribosyl)-6,7-dihydro-6,7-dihydroxy-6/7-methylimidazo-[2,3-b]purine-9(8)one isomers; MG-H1, methylglyoxal-derived hydroimidazolone, *N*^δ^-(5-hydro-5-methyl-4-imidazolone-2-yl)-ornithine; RAGE, receptor for advanced glycation adducts; and UPR, unfolded protein response.

## References

[1] International-Maillard-Reaction-Society, (IMARS). https://www.imarsonline.com/.

[2] Rabbani N., Thornalley P.J. (2012). Glycation research in Amino Acids: A place to call home. Amino Acids.

[3] Rabbani N., Thornalley P.J. (2012). Methylglyoxal, glyoxalase 1 and the dicarbonyl proteome. Amino Acids.

[4] Rabbani N., Thornalley P.J. (2021). Protein glycation—Biomarkers of metabolic dysfunction and early-stage decline in health in the era of precision medicine. Redox Biol..

[5] Thornalley P.J., Battah S., Ahmed N., Karachalias N., Agalou S., Babaei-Jadidi R., Dawnay A. (2003). Quantitative screening of advanced glycation endproducts in cellular and extracellular proteins by tandem mass spectrometry. Biochem. J..

[6] Ahmed M.U., Thorpe S.R., Baynes J.W. (1986). Identification of Nε-carboxymethyl-lysine as a degradation product of fructoselysine in glycated protein. J. Biol.Chem..

[7] Sell D.R., Monnier V.M. (1989). Structure elucidation of a senescence crosslink from human extracellular matrix. Implication of pentoses in the aging process. J. Biol.Chem..

[8] Ahmed N., Dobler D., Dean M., Thornalley P.J. (2005). Peptide mapping identifies hotspot site of modification in human serum albumin by methylglyoxal involved in ligand binding and esterase activity. J. Biol. Chem..

[9] Thornalley P.J., Waris S., Fleming T., Santarius T., Larkin S.J., Winklhofer-Roob B.M., Stratton M.R., Rabbani N. (2010). Imidazopurinones are markers of physiological genomic damage linked to DNA instability and glyoxalase 1-associated tumour multidrug resistance. Nucleic Acids Res..

[10] Rabbani N., Xue M., Thornalley P.J. (2016). Methylglyoxal-induced dicarbonyl stress in aging and disease: First steps towards glyoxalase 1-based treatments. Clin. Sci..

[11] Ban I., Sugawa H., Nagai R. (2022). Protein Modification with Ribose Generates N(δ)-(5-hydro-5-methyl-4-imidazolone-2-yl)-ornithine. Int. J. Mol. Sci..

[12] Thornalley P.J., Langborg A., Minhas H.S. (1999). Formation of glyoxal, methylglyoxal and 3-deoxyglucosone in the glycation of proteins by glucose. Biochem. J..

[13] Ahmed N., Thornalley P.J. (2002). Chromatographic assay of glycation adducts in human serum albumin glycated In Vitro by derivatisation with aminoquinolyl-N-hydroxysuccimidyl-carbamate and intrinsic fluorescence. Biochem. J..

[14] Schalkwijk C., Stehouwer C.D. (2019). Methylglyoxal, a highly reactive dicarbonyl compound, in diabetes, its vascular complications and other age-related diseases. Physiol. Rev..

[15] Monnier V.M., Cerami A. (1981). Nonenzymatic browning In Vivo: Possible process for aging of long lived proteins. Science.

[16] Hellwig M., Henle T. (2014). Baking, ageing, diabetes: A short history of the Maillard reaction. Angew. Chem. (Int. Ed. Engl.).

[17] Delgado-Andrade C., Fogliano V. (2018). Dietary Advanced Glycosylation End-Products (dAGEs) and Melanoidins Formed through the Maillard Reaction: Physiological Consequences of their Intake. Annu. Rev. Food Sci. Technol..

[18] Sies H., Berndt C., Jones D.P. (2017). Oxidative Stress. Annu. Rev. Biochem..

[19] Rabbani N., Thornalley P.J. (2015). Dicarbonyl stress in cell and tissue dysfunction contributing to ageing and disease. Biochem. Biophys. Res. Commun..

[20] Gallet X., Charloteaux B., Thomas A., Braseur R. (2000). A fast method to predict protein interaction sites from sequences. J. Mol. Biol..

[21] Al-Motawa M.S., Abbas H., Wijten P., de la Fuente A., Xue M., Rabbani N., Thornalley P.J. (2020). Vulnerabilities of the SARS-CoV-2 Virus to Proteotoxicity—Opportunity for Repurposed Chemotherapy of COVID-19 Infection. Front. Pharmacol..

[22] Rabbani N., Al-Motawa M., Thornalley P.J. (2020). Protein Glycation in Plants—An Under-Researched Field with Much Still to Discover. Int. J. Mol. Sci..

[23] Rabbani N., Xue M., Thornalley P.J. (2021). Dicarbonyl stress, protein glycation and the unfolded protein response. Glycoconj. J..

[24] Irshad Z., Xue M., Ashour A., Larkin J.R., Thornalley P.J., Rabbani N. (2019). Activation of the unfolded protein response in high glucose treated endothelial cells is mediated by methylglyoxal. Sci. Rep..

[25] Ashour A., Xue M., Al-Motawa M., Thornalley P.J., Rabbani N. (2020). Glycolytic overload-driven dysfunction of periodontal ligament fibroblasts in high glucose concentration, corrected by glyoxalase 1 inducer. BMJ Open Diabetes Res. Care.

[26] Zender L., Xue W., Zuber J., Semighini C.P., Krasnitz A., Ma B., Zender P., Kubicka S., Luk J.M., Schirmacher P. (2008). An Oncogenomics-Based In Vivo RNAi Screen Identifies Tumor Suppressors in Liver Cancer. Cell.

[27] Donnellan L., Young C., Simpson B.S., Dhillon V.S., Costabile M., Hoffmann P., Fenech M., Deo P. (2022). Methylglyoxal Impairs Sister Chromatid Separation in Lymphocytes. Int. J. Mol. Sci..

[28] Rabbani N., Thornalley P.J. (2022). Emerging Glycation-Based Therapeutics—Glyoxalase 1 Inducers and Glyoxalase 1 Inhibitors. Int. J. Mol. Sci..

[29] Alhujaily M., Abbas H., Xue M., de la Fuente A., Rabbani N., Thornalley P.J. (2021). Studies of Glyoxalase 1-Linked Multidrug Resistance Reveal Glycolysis—Derived Reactive Metabolite, Methylglyoxal, Is a Common Contributor in Cancer Chemotherapy Targeting the Spliceosome. Front. Oncol..

[30] Thornalley P.J., Edwards L.G., Kang Y., Wyatt C., Davies N., Ladan M.J., Double J. (1996). Antitumour activity of S-p- bromobenzylglutathione cyclopentyl diester In Vitro and In Vivo: Inhibition of glyoxalase I and induction of apoptosis. Biochem. Pharmacol..

[31] Rabbani N., Xue M., Weickert M.O., Thornalley P.J. (2018). Multiple roles of glyoxalase 1-mediated suppression of methylglyoxal glycation in cancer biology—Involvement in tumour suppression, tumour growth, multidrug resistance and target for chemotherapy. Semin. Cancer Biol..

[32] Lo T.W.C., Thornalley P.J. (1992). Inhibition of proliferation of human leukemia 60 cells by diethyl esters of glyoxalase inhibitors in vitro. Biochem. Pharmacol..

[33] Thornalley P.J. (1995). Advances in glyoxalase research. Glyoxalase expression in malignancy, anti-proliferative effects of methylglyoxal, glyoxalase I inhibitor diesters and S -D-lactoylglutathione, and methylglyoxal-modified protein binding and endocytosis by the advanced glycation endproduct receptor. Crit. Rev. Oncol. Haematol..

[34] Jandial R., Neman J., Lim P.P., Tamae D., Kowolik C.M., Wuenschell G.E., Shuck S.C., Ciminera A.K., De Jesus L.R., Ouyang C. (2018). Inhibition of GLO_1_ in Glioblastoma Multiforme Increases DNA-AGEs, Stimulates RAGE Expression, and Inhibits Brain Tumor Growth in Orthotopic Mouse Models. Int. J. Mol. Sci..

[35] Donnellan L., Young C., Simpson B.S., Acland M., Dhillon V.S., Costabile M., Fenech M., Hoffmann P., Deo P. (2022). Proteomic Analysis of Methylglyoxal Modifications Reveals Susceptibility of Glycolytic Enzymes to Dicarbonyl Stress. Int. J. Mol. Sci..

[36] Galligan J.J., Wepy J.A., Streeter M.D., Kingsley P.J., Mitchener M.M., Wauchope O.R., Beavers W.N., Rose K.L., Wang T., Spiegel D.A. (2018). Methylglyoxal-derived posttranslational arginine modifications are abundant histone marks. Proc. Natl. Acad. Sci. USA.

[37] Perez-Riverol Y., Bai J., Bandla C., García-Seisdedos D., Hewapathirana S., Kamatchinathan S., Kundu D.J., Prakash A., Frericks-Zipper A., Eisenacher M. (2022). The PRIDE database resources in 2022: A hub for mass spectrometry-based proteomics evidences. Nucleic Acids Res..

[38] Ahmed N., Argirov O.K., Minhas H.S., Cordeiro C.A., Thornalley P.J. (2002). Assay of advanced glycation endproducts (AGEs): Surveying AGEs by chromatographic assay with derivatisation by aminoquinolyl-N-hydroxysuccimidyl-carbamate and application to Nε-carboxymethyl-lysine- and Nε-(1-carboxyethyl)lysine-modified albumin. Biochem. J..

[39] Rabbani N., Ashour A., Thornalley P.J. (2016). Mass spectrometric determination of early and advanced glycation in biology. Glycoconj. J..

[40] Gahete M.D., del Rio-Moreno M., Camargo A., Alcala-Diaz J.F., Alors-Perez E., Delgado-Lista J., Reyes O., Ventura S., Perez-Martínez P., Castaño J.P. (2018). Changes in Splicing Machinery Components Influence, Precede, and Early Predict the Development of Type 2 Diabetes: From the CORDIOPREV Study. EBioMedicine.

[41] Newman J.R.B., Conesa A., Mika M., New F.N., Onengut-Gumuscu S., Atkinson M.A., Rich S.S., McIntyre L.M., Concannon P. (2017). Disease-specific biases in alternative splicing and tissue-specific dysregulation revealed by multitissue profiling of lymphocyte gene expression in type 1 diabetes. Genome Res..

[42] Stevens M., Oltean S. (2016). Alternative Splicing in CKD. J. Am. Soc. Nephrol..

[43] Raj T., Li Y.I., Wong G., Humphrey J., Wang M., Ramdhani S., Wang Y.-C., Ng B., Gupta I., Haroutunian V. (2018). Integrative transcriptome analyses of the aging brain implicate altered splicing in Alzheimer’s disease susceptibility. Nat. Genet..

[44] Rabbani N., Xue M., Thornalley P.J. (2022). Hexokinase-2-Linked Glycolytic Overload and Unscheduled Glycolysis—Driver of Insulin Resistance and Development of Vascular Complications of Diabetes. Int. J. Mol. Sci..

[45] Stratmann B. (2022). Dicarbonyl Stress in Diabetic Vascular Disease. Int. J. Mol. Sci..

[46] Ramasamy R., Shekhtman A., Schmidt A.M. (2022). The RAGE/DIAPH1 Signaling Axis & Implications for the Pathogenesis of Diabetic Complications. Int. J. Mol. Sci..

[47] Snelson M., Lucut E., Coughlan M.T. (2022). The Role of AGE-RAGE Signalling as a Modulator of Gut Permeability in Diabetes. Int. J. Mol. Sci..

[48] Oshima Y., Harashima A., Munesue S., Kimura K., Leerach N., Goto H., Tanaka M., Niimura A., Hayashi K., Yamamoto H. (2022). Dual Nature of RAGE in Host Reaction and Nurturing the Mother—Infant Bond. Int. J. Mol. Sci..

[49] Inagi R. (2022). Organelle Stress and Metabolic Derangement in Kidney Disease. Int. J. Mol. Sci..

[50] Rabbani N. (2022). AGEomics Biomarkers and Machine Learning-Realizing the Potential of Protein Glycation in Clinical Diagnostics. Int. J. Mol. Sci..

[51] Linkens A.M.A., van Best N., Niessen P.M., Wijckmans N.E.G., de Goei E.E.C., Scheijen J., van Dongen M., van Gool C., de Vos W.M., Houben A. (2022). A 4-Week Diet Low or High in Advanced Glycation Endproducts Has Limited Impact on Gut Microbial Composition in Abdominally Obese Individuals: The deAGEing Trial. Int. J. Mol. Sci..

[52] Maillard L.C. (1912). Action des acides amines sur les sucres: Formation des melanoidines par voie methodique. Compt. Rend. Hebd. Seances Acad. Sci..

[53] Neuberg C. (1913). The destruction of lactic aldehyde and methylglyoxal by animal organs. Biochem Z.

[54] Dakin H.D., Dudley H.W. (1913). An enzyme concerned with the formation of hydroxy acids from ketonic aldehydes. J. Biol. Chem..

[55] Kuhn R., Weygand F. (1937). The Amadori rearrangement. Ber. Dtsch. Chem. Ges..

[56] Hodge J.E. (1953). Dehydrated foods: Chemistry of browning reactions in model systems. J. Agric. Food Chem..

[57] Kato H. (1960). Studies on browning reactions between sugars and amino compounds. V. Isolation and characterisation of new carbonyl compounds, 3-deoxyglucosones formed from N-glycosides and their significance for browning reaction. Bull. Agric. Chem. Soc. Jpn..

[58] Rahbar S. (1968). An abnormal hemoglobin in red cells of diabetics. Clin. Chim. Acta.

[59] Hayashi T., Namiki M. (1980). Formation of two-carbon sugar fragments at an early stage of the browning reaction of sugar and amine. Agric. Biol. Chem..

[60] Pongor S., Ulrich P.C., Benesath F.A., Cerami A. (1984). Aging of protiens: Isolation and identification of a fluorescent chromophore from the reaction of polypeptides with glucose. Proc. Natl. Acad. Sci. USA.

[61] Schmidt A.M., Vianna M., Gerlach M., Brett J., Ryan J., Kao J., Esposito C., Hegarty H., Hurley W., Clauss M. (1992). Isolation and characterization of two binding proteins for advanced glycosylation endproducts from bovine lung which are present on the endothelial cell surface. J. Biol. Chem..

[62] Bolton W.K., Cattran D.C., Williams M.E., Adler S.G., Appel G.B., Cartwright K., Foiles P.G., Freedman B.I., Raskin P., Ratner R.E. (2004). Randomized trial of an inhibitor of formation of advanced glycation end products in diabetic nephropathy. Am. J. Nephrol..

[63] Freedman B.I., Wuerth J.P., Cartwright K., Bain R.P., Dippe S., Hershon K., Mooradian A.D., Spinowitz B.S. (1999). Design and baseline characteristics for the aminoguanidine clinical trial in overt type 2 diabetic nephropathy (ACTION II). Control. Clin. Trials.

[64] Henle T., Walter A., Haeáner R., Klostermeryer H. (1994). Detection and identification of a protein-bound imidazolone resulting from the reaction of arginine residues and methylglyoxal. Z. Lebensm. Unters. Forsch..

[65] Delpierre G., Rider M.H., Collard F., Stroobant V., Vanstapel F., Santos H., Van Schaftingen E. (2000). Identification, cloning, and heterologous expression of a mammalian fructosamine-3-kinase. Diabetes.

[66] Morcos M., Du X., Pfisterer F., Hutter H., Sayed A.A.R., Thornalley P., Ahmed N., Baynes J., Thorpe S., Kukudov G. (2008). Glyoxalase-1 prevents mitochondrial protein modification and enhances lifespan in *Caenorhabditis elegans*. Aging Cell.

[67] Xue M., Weickert M.O., Qureshi S., Ngianga-Bakwin K., Anwar A., Waldron M., Shafie A., Messenger D., Fowler M., Jenkins G. (2016). Improved glycemic control and vascular function in overweight and obese subjects by glyoxalase 1 inducer formulation. Diabetes.

[68] Ahmed U., Anwar A., Savage R.S., Thornalley P.J., Rabbani N. (2016). Protein oxidation, nitration and glycation biomarkers for early-stage diagnosis of osteoarthritis of the knee and typing and progression of arthritic disease. Arthritis Res. Ther..

[69] Xing H., Yaylayan V. (2022). Insight into Isomeric Diversity of Glycated Amino Acids in Maillard Reaction Mixtures. Int. J. Mol. Sci..

[70] Linden T., Forsback G., Deppisch R., Henle T., Wieslander A. (1998). 3-Deoxyglucosone, a promoter of advanced glycation end products in fluids for peritoneal dialysis. Perit. Dial. Int..

[71] Auditore A., Gensberger-Reigl S., Pischetsrieder M. (2022). In Vitro Reactivity of the Glucose Degradation Product 3,4-Dideoxyglucosone-3-ene (3,4-DGE) towards Abundant Components of the Human Blood Circulatory System. Int. J. Mol. Sci..

